# Metabonomic Evaluation of ZHENG Differentiation and Treatment by Fuzhenghuayu Tablet in Hepatitis-B-Caused Cirrhosis

**DOI:** 10.1155/2012/453503

**Published:** 2012-05-29

**Authors:** Shujun Sun, Jianye Dai, Wenyu Wang, Huijuan Cao, Junwei Fang, Yi Yang Hu, Shibing Su, Yongyu Zhang

**Affiliations:** ^1^Research Center for Traditional Chinese Medicine and Systems Biology, Shanghai University of Traditional Chinese Medicine, 1200 Cailun Road, Pudong, Shanghai 201203, China; ^2^Institute of Liver Diseases, Shuguang Hospital, Key Laboratory of Liver and Kidney Diseases of Ministry of Education, Shanghai University of Traditional Chinese Medicine, Shanghai 201203, China; ^3^Research Center for Traditional Chinese Medicine Complexity System, Shanghai University of Traditional Chinese Medicine, 1200 Cailun Road, Pudong, Shanghai 201203, China

## Abstract

In Traditional Chinese Medicine (TCM), treatment based on ZHENG (also called TCM syndrome and pattern) differentiation has been applied for about 3 thousand years, while there are some difficulties to communicate with western medicine. In the present work, metabonomic methods were utilized to differentiate ZHENG types and evaluate the therapeutic efficiency of Fuzhenghuayu (FZHY) tablet in hepatitis-B-caused cirrhosis (HBC). Urine samples of 12 healthy volunteers (control group, CG) and 31 HBC patients (HBCG) were analyzed by gas chromatography mass spectrometry (GC/MS) and multivariate statistical analysis. The significantly changed metabolites between CG and HBCG were selected by PLS-DA loading plot analysis. Moreover, 4 ZHENGs were differentiated mutually, suggesting that there was urine metabolic material basis in ZHENG differentiation. The efficiency of FZHY tablet on subjects with spleen deficiency with dampness encumbrance syndrome (SDDES) and liver-kidney yin deficiency syndrome (LKYDS) was better than that of other syndromes. The efficiency of FZHY treatment based on ZHENG differentiation indicated that accurately ZHENG differentiating could guide the appropriate TCM treatment in HBC.

## 1. Introduction

Cirrhosis and its complications are one of the main causes of mortality [1] especially for individuals aged 45 to 54 years [2]. Hepatitis B virus (HBV) infection is one of the most common viral infections in humans. Approximately 350 million people have been chronically infected by HBV [3], and around 20% to 30% of them will result in cirrhosis [4]. To date, the definite and indefinite duration treatments with interferon and nucleotide analogue, respectively, are two first line strategies in western medicine [4]. Facing the sustained high morbidity and mortality, new effective therapeutic protocols of HBC are imperative. As a holistic and multitarget approach, TCM has shown some advantages on the treatment of those complicated chronic diseases [5–7].

With increasing attentions paid to TCM, many researches [5–7] about the curative effect of Chinese medicinal and formulas were conducted but lack direction of TCM theory. TCM is a large and complex system, and ZHENG differentiation and treatment (Bian Zheng Lun Zhi) is one of its essences. To further explaination, the diagnosis that guides treatment of TCM is called ZHENG (TCM syndrome or pattern), a temporary state at one time and which is defined by symptoms and signs. It could be assessed by four diagnostic methods (looking, listening and smelling, asking, and touching) [8]. And the same disease can usually manifest in different syndromes, so patients with different ZHENG types may be treated by different rules and therapeutic regimen. Based on the holistic and systemic characteristics of ZHENG, metabonomics [9], genomics [10], proteomics [11], and the integration of them [12] were introduced to the research of ZHENG differentiation.

The marketed Fuzhenghuayu tablet is a TCM prescription including *Radix Salvia miltiorrhizae, Cordyceps mycelia extract, Semen Persicae, Gynostemma pentaphyllum Mak, Pollen Pini *and* Fructus schisandrae chinensis*. The recipe composition was directed by the therapeutic method of invigorating blood transforming stasis and boosting essence supplementing deficiency [13]. In the present study, urine Metabonomics [14] based on gas chromatography mass spectrometry (GC/MS) and multivariate statistical techniques was utilized to differentiate four ZHENG types of HBC in the molecular level and evaluate the therapeutic effects of FZHY tablet for different ZHENG types. To our knowledge, this study is the first report of urinary Metabonomics method used to investigate the therapeutic effects of FZHY tablet for different ZHENGs.

## 2. Materials and Methods

### 2.1. Subjects and Experiment Design

Twelve healthy volunteers and 31 patients from Shanghai Shuguang Hospital (Shanghai, China) were enrolled in the study. The healthy volunteers without any treatment were considered as CG. All of patients were affected with hepatitis-B-caused cirrhosis (HBC) and regarded as disease group (HBCG). The patients were classified into 4 ZHENG types, including spleen deficiency with dampness encumbrance syndrome (SDDES, *n* = 7), liver-gallbladder dampness-heat syndrome (LGDHS, *n* = 7), liver-kidney yin deficiency syndrome (LKYDS, *n* = 10), and blood stasis syndrome (BSS, *n* = 7). All of them were treated with the same formula by oral administration. Then metabonomic detection and analysis was performed to evaluate the therapeutic effect on HBC patients with different ZHENG types. The clinical study was approved by the local ethics committee and all of the recruited persons were given informed consent. Diagnosis standard of cirrhosis is referred to “Chronic hepatitis B prevention and treatment guidelines.” [15]. And all cases of HBC caused by other factors such as hepatitis C infection, alcohol consumption, and usage of drugs with hepatotoxicity were ruled out before all the subjects entered the study. The TCM ZHENG types were identified by three chief or deputy physicians, according to “evaluation criteria of the clinical diagnosis, drug efficacy, and ZHENG differentiation for cirrhosis (pilot program)” [16]. The study was performed in accordance with the principles contained in the Declaration of Helsinki.

### 2.2. Chemicals and Drugs

Ethyl chloroformate (ECF), pyridine, anhydrous ethanol, sodium hydroxide, chloroform, and anhydrous sodium sulfate were analytical grade from China National Pharmaceutical Group Corporation (Shanghai, China). L-2-chlorophenylalanine (Shanghai Intechem Tech. Co. Ltd., China) was used as an internal quality standard which was prepared in the ultrapure water from a Milli-Q system (Millipore, USA). FZHY tablets were provided by Shanghai Huanghai Pharmaceutical Co., Ltd.

### 2.3. Sample Collection and Preparation

Urina sanguinis was collected from 12 healthy subjects and 31 HBC patients when they were enrolled in the study. And after 12 and 24 weeks of treatment the patients were asked for urina sanguinis again. Urine samples were stored at −80°C until GC-MS assay.

All these samples were thawed in ice water bath and vortex-mixed before analysis. Each 600 *μ*L aliquot of standard mixture or urine sample was added to a screw tube. After adding 100 *μ*L of l-2-chlorophenylalanine (0.1 mg mL^−1^), 400 *μ*L of anhydrous ethanol, and 100 *μ*L of pyridine to the urine sample, 50 *μ*L of ECF was added for first derivatization at 20.0 ± 0.1°C. The pooled mixtures were sonicated at 40 kHz for 60 s. Subsequently, extraction was performed using 300 *μ*L of chloroform, with the aqueous layer pH carefully adjusted to 9-10 using 100 *μ*L of NaOH (7 mol L^−1^). The derivatization procedure was repeated with the addition of 50 *μ*L ECF into the aforementioned products. After the two successive derivatization steps, the overall mixtures were vortexed for 30 s and centrifuged for 3 min at 3000 rpm. The aqueous layer was aspirated off, while the remaining chloroform layer containing derivatives was isolated and dried with anhydrous sodium sulfate and subsequently subjected to GC-MS. The derivatization method referred to [17].

### 2.4. Data Acquisition

All GC-MS analyses were performed by a mass spectrometer 5975B (Agilent technologies, USA) coupled to an Agilent 6890 (Agilent technologies, USA) gas chromatography instrument. In the gas chromatographic system, a catabletary column (Agilent J&W DB-5 ms Ultra Inert 30 m × 0.25 mm, film thickness 0.25 *μ*m) was used. Helium carrier gas was used at a constant flow rate of 1.0 mL ∗ min^−1^. One *μ*L of derivatized samples was injected into the GC/MS instrument, and splitless injection mode was used. To acquire a well separation, the column temperature was initially maintained at 80°C for 2 min and then increased from 80 to 140°C at the rate of 10°C/min for 6 min. Then, the column temperature was increased to 240°C at the rate of 4°C/min for 25 min. After that, the column temperature was increased to 280°C at the rate of 10°C/min for 4 min and held for 3 min. The temperatures of the injection port, the interface, and source temperature were set at 280°C, 260°C, and 230°C, respectively. The measurements were made with electron impact ionization (70 eV) in the full scan mode (m/z 30–550). The solvent posttime was set to 5 min.

### 2.5. Data Analysis

Due to experimental variations and column aging, shifts in retention time between fingerprints occur. When the total ion current chromatograms (TICs) were obtained, peak-alignment or warping techniques are commonly applied to compensate for minor shifts in retention times. Thus, in the subsequently data processing, the same variable manifested synchronous information in every profile. So all the GC-MS raw files after being converted to CDF format via the software come with Agilent MSD workstation, and were subsequently processed by the XCMS toolbox (http://metlin.scripps.edu/download/) using XCMS's default settings with the following exceptions: xcmsSet (full width at half-maximum: fwhm = 5; S/N cutoff value: snthresh = 10, max = 15), and group (bw = 5). The resulting table (CSV file) was exported into Microsoft Excel (Microsoft Inc., USA), where normalization was performed prior to multivariate analyses. The resulting three-dimensional matrix involving peak index (RT-m/z pair), sample names (observations), and normalized peak area percent was introduced into Simca-P 11.5 Software package (Umetrics, Umea, Sweden) for partial least squares-discriminate analysis (PLS-DA). Differential variables between CG and HBCG were generated from loadings plot. To find the influential metabolites responsible for the separation, we calculated the variable importance for the projection (VIP) values [18]. Variables with VIP values exceeding 1.5 were first selected. In a second step, those variables were further compared by Mann-Whitney *U*-test to confirm the changed metabolites in SPSS 17.0 (SPSS, Chicago, IL, USA) with the threshold of *P* value set at 0.05. Those variables, then, were identified by searching in NIST 2005 database and verified by standards. References and the Kyoto Encyclopedia of Genes and Genomes (KEGG) (http://www.genome.ad.jp/kegg/) were based to give the biochemical interpretation of changed metabolites affected by HBC.

## 3. Results

### 3.1. Metabolic Profiles of Cirrhosis Patients and Healthy Control

**Figure 1:**
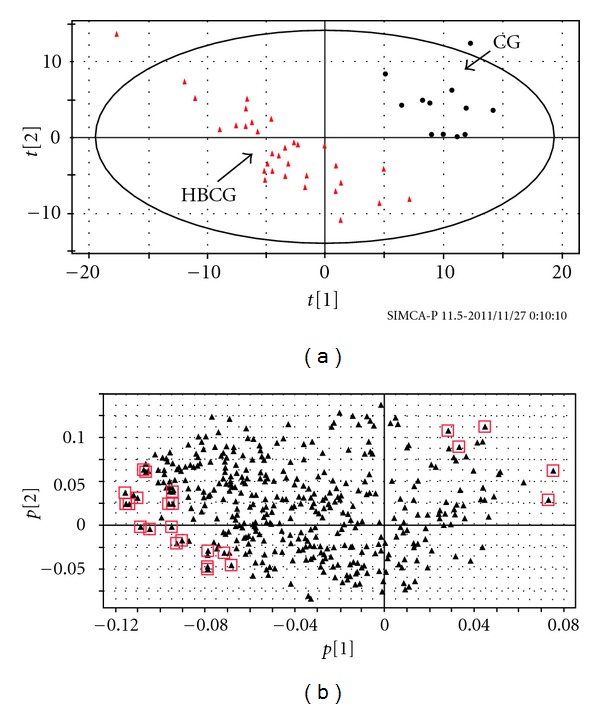
(a) PLS-DA score plot between CG and HBCG. Black dots and red triangles refer to healthy subjects and hepatitis-B-caused Cirrhosis subjects, respectively. (b) PLS-DA loading plot from HBCG and CG.

One *μ*L aliquots of supernatants of all the urine samples, after a two-step derivatization, extraction and dryness, were injected into GC/MS for analysis with the method described previously. PLS-DA analysis was employed to discriminate HBCG and CG, and the score plot with *R* ^2^ *Y* = 0.888 and *Q* ^2^ *Y* = 0.792 is shown in Figure 1(a). In this map, HBCG could be absolutely separated from healthy group. The results might demonstrate that the urine metabolic profiles had changed significantly.

A loading plot was constructed to indicate the most influential variables according to their respective contributions to the discrimination between the 2 groups (Figure 1(b)). The further away from the main cluster, the greater influence the variables have on the PLS-DA scores plot. Every variable could be identified by the measured m/z value and NIST database. The metabolites' names, corresponding VIP values, and changed trend compared with the healthy group are presented in Table 1, simultaneously.

### 3.2. Biochemical Interpretation

#### 3.2.1. Disorder of Immunity

**Figure 2:**
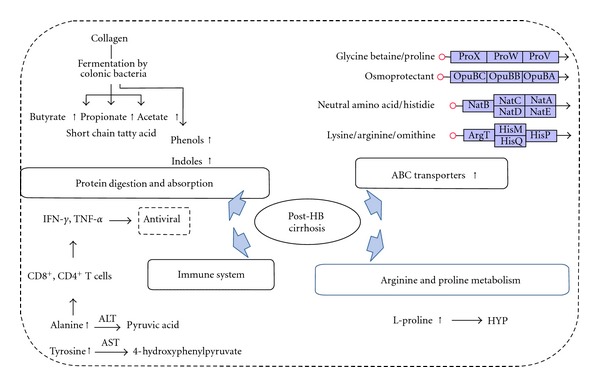
HBC-related pathway observed in this research.

Alanine and tyrosine, which are the substrates of alanine transaminase (ALT) and aspartate aminotransferase (AST), respectively, were upregulated in HBCG. And ALT and AST will increase when activated CD4^+^ and CD8^+^ lymphocytes recognize various HBV-derived proteins located on the surface of infected hepatocytes [19] (summarized in Figure 2). So the increase of alanine and tyrosine might suggest that HBC was correlated with the disorder of immune system, which was in agreement with the previous report that chronic HBV infection develops in the setting of impaired immune reactions or a relatively tolerant immune system status [20].

#### 3.2.2. Energy Metabolism

Alanine and proline are precursors of pyruvate which can convert to acetyl-coenzyme A (Acetyl-CoA) and is the main input for a series of reactions known as TCA cycle. The increased level of alanine and proline in HBCG might indicate that HBCG relieves the inhibition of proline iminopeptidase (PIP) and activates the biosynthesis of pyruvate to increase carbohydrate catabolism [20].

#### 3.2.3. ABC Transporters

The significantly changed metabolites in this study, proline, lysine, and alanine, participated the pathway of ABC transporters, which was found in database KEGG. Liver is the most active site of cholesterol metabolism, and the content of cholesterol is closely correlated with cirrhosis [21]. However, ABC transporters play an important role in secretion of cholesterol from liver into bile [22]. The three changed amino acids might suggest that HBC would be correlated with the dysfunction of ABC transporters, which was in accordance with the literature [23].

#### 3.2.4. Protein Digestion and Absorption

The contents of precollagen type III and collagen type IV in cirrhosis subjects are higher than normal ones, and they were reported as two of the main factors for hepatitis fibrosis and cirrhosis [23]. Butyrate, propionate, acetate, phenol, and indole are the products of collagens after fermentation by colonic bacteria. Those compounds that were detected increased compared with healthy group in this research. Among them butyrate, propionate, and acetate were retrieved in form of their acid which were listed as butanoic acid, propanoic acid, and acetic acid in Table 1. The results might prompt that the collagens in subjects of this research have been improved, meaning that they may have been affected with HBC.

In addition, Hydroxyproline, a product of proline hydroxylation, is a common used biomarker of fibrosis or cirrhosis in animal experiments. In tissue of animals with cirrhosis, the content of hydroxyproline is much higher than healthy group [5, 6], which manifested in the increase of proline in urine samples in this test. We have detected several small molecules including alanine, tyrosine, butanoic acid, propanoic acid, and acetic acid dovetailing with clinical biomarkers ALT, AST, precollagen type III, and collagen type IV in this research, which suggests metabonomic technology or further studies could help diagnose HBC.

### 3.3. ZHENG Differentiation

**Figure 3:**
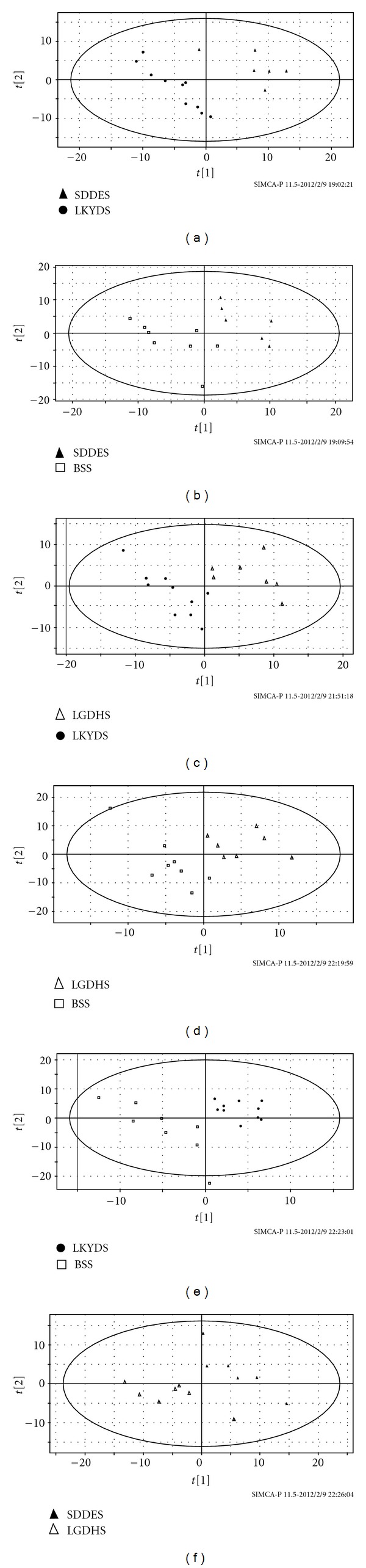
Score plot of PLS-DA for comparison among 4 ZHENG types. SDDES compared to LKYDS (a); SDDES compared to BSS (b); LGDHS compared to LKYDS (c); LGDHS compared to BSS (d); LKYDS compared to BSS (e); SDDES compared to LGDHS (f).

Four ZHENG types were distinguished by PLS-DA analysis. The model information is shown in Table 2, and six maps of score plot are presented in Figure 3. The results prompt that ZHENG differentiation in TCM may be based on objective material, not only on practitioners' experience.

### 3.4. Efficiency of FZHY Tablet

**Figure 4:**
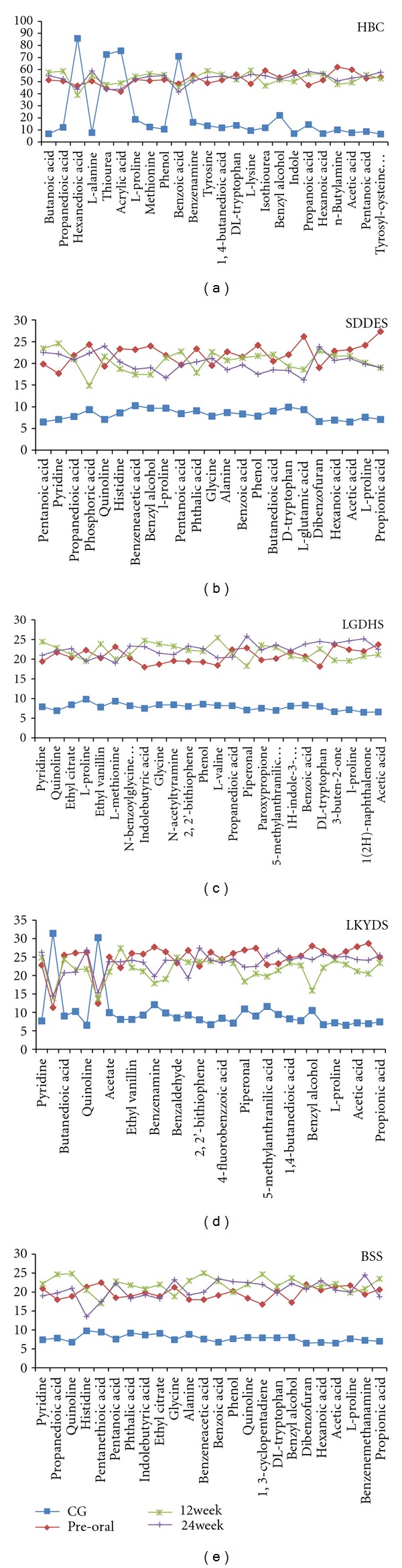
Five maps revealed the therapeutic effect of FZHY tablet for hepatitis-B-caused cirrhosis (HBC) and four TCM ZHENG types, respectively: spleen deficiency with dampness encumbrance syndrome (SDDES), liver-gallbladder dampness-heat syndrome (LGDHS), liver-kidney yin deficiency syndrome (LKYDS), and blood stasis syndrome (BSS), by the changing trend of significantly differential metabolites. CG is short for control group; preoral in each group stands for information before intervention of FZHY tablet; 12th week means effects after 12-week intervention by FZHY tablet; 24th week means effects after 24-week intervention. The *x*-axis represented the changed metabolites, and the *y*-axis was average rank in Mann-Whitney *U*-test, representing the contents of metabolites.

The significantly changed metabolites of each group HBC patients from the healthy subjects have been selected. And the potential biomarkers of HBCG were previously listed in Table 1. The four TCM ZHEGNs' potential biomarkers are not summarized in tables but can be found on *x*-axis of Figure 4. The reversions of these metabolites were based on to evaluate the therapeutic effect of FZHY tablet. Consequently, we found that there were no significantly reversed potential biomarkers for all the subjects of HBC at both 12th week and 24th week. While for LSYDS at 12th week, most influential metabolites reversed, and at 24th week, the reversion of potential biomarkers showed good efficiency of FZHY for SDDES, as manifested in Figure 4 (LKYDS and SDDES). As we can see, most metabolites for LGDHS and BBS got further away from the healthy group than pre-oral of FZHY.

## 4. Discussion

The potential biomarkers that discriminate HBCG and CG were found by PLS-DA loading plot analysis. After retrieving literatures and the database KEGG, it was supposed that HBC might correlate with the disorder of immune metabolism, energy metabolism, ABC transporters, and protein digestion and absorption. The ZHENG differentiation of HBC demonstrated that a disease might be divided into more than one pattern. Different metabolic profiles or different phenotypes probably arise from disparate pathogenesis and etiological factors. Consequently, every ZHENG should be treated differently, which was in accordance with the theory of ZHENG differentiation and treatment [24].

The results showed that subjects with deficiency syndrome (SDDES and LKYDS) are more susceptible for FZHY tablet, which was in accordance with “boosting essence supplementing deficiency” in the principles of recipe composition. At 24th week, subjects with LKYDS did not appear the effects as good as that of 12th week, which may be interpreted by the dynamic and developmental characteristics of disease. The patients were diagnosed with LKYDS when enrolled, but after 12 weeks their ZHENG might have changed. So the treatment rules should be changed correspondingly, which precisely manifested the personalized medicine. In respect of that subjects with LGDHS and BSS still had no signs of recovery during the 24-week treatment, those two ZHENG types seemed not suitable for FZHY tablet. So the correspondence between ZEHNG and formula, called “fang zheng dui ying” in TCM, is very important [25]. And it is the main treatment principle after ZHENG differentiation.

To acquire results with high reliability and accuracy, large amount of samples should be collected. And after the multicenter and multiregional trial validation, biomarkers could only be transformed into clinical applications. In addition, if Metabonomics is validated by other “omics” or biochemical methods, it would be more convincing.

## 5. Conclusion

Subjects with HBC were distinguished from the healthy control with the method of Metabonomics based on GC/MS analysis and multivariate statistical techniques. The four ZHENGs in this study were also classified by PLS-DA. Without ZHENG differentiation, the efficiency of FZHY tablet for patients with HBC was not significant, through the holistic evaluating approach. However if the objects of treatment aim at subjects with spleen deficiency with dampness encumbrance syndrome or liver-kidney yin deficiency syndrome, the therapeutic effects would be increased remarkably. And at ones with liver-gallbladder dampness-heat syndrome, and blood stasis syndrome, within 24 weeks not any effects could be observed. As a result, the treatment effect of FZHY tablet indicated that accurately ZHENG differentiation could guide the appropriate TCM treatment in HBC. And this study indicated that Metabonomics technology can be utilized to evaluate the therapeutic effect of TCM recipes based on ZHENG differentiation and Treatment.

## Figures and Tables

**Table 1:** Identification results and the changed trend of differential metabolites of hepatitis-B-caused Cirrhosis subjects compared to healthy group.

Number	Metabolites	RT (min)	VIP	Changing trend compared with HG
1	Butanoic acid	5.29	2.25	↑**
2	Propanedioic acid	5.98	1.81	↑**
3	Hexanedioic acid	6.03	1.97	↓**
4	L-Alanine	8.06	2.28	↑**
5	Thiourea	9.75	1.73	↓**
6	acrylic acid	12.91	1.55	↓**
7	L-Proline	16.9	2.12	↑**
8	Methionine	17.8	1.57	↑**
9	Phenol	27.87	2.08	↑**
10	Benzoic acid	31.71	1.59	↓**
11	Benzenamine	31.79	1.97	↑**
12	Tyrosine	32.49	1.75	↑**
13	1,4-Butanedioic acid	34.41	1.92	↑**
14	DL-Tryptophan	34.44	1.83	↑**
15	L-Lysine	34.75	2.11	↑**
16	Isothiourea	34.78	2.04	↑**
17	Benzyl alcohol	35.08	1.61	↑**
18	Indole	36.21	2.03	↑**
19	Propanoic acid	36.4	1.64	↑**
20	Hexanoic acid	36.71	2.26	↑**
21	n-Butylamine	36.78	1.88	↑**
22	Acetic acid	36.81	1.69	↑**
23	Pentanoic acid	36.89	1.64	↑**
24	Tyrosyl-cysteine methyl ester	37.37	2.04	↑**

The levels of differential metabolites were labeled with (↓) downregulated and (↑) upregulated (**P < 0.01).

**Table 2:** Model information of PLS-DA for comparison of 4 ZHENGs with each other.

Model	Amount of components	*R* ^2^ *Y*	*Q* ^2^ *Y*
SDDES and LKYDS	3	0.969	0.462
SDDES and BSS	3	0.922	0.106
LGDHS and LKYDS	3	0.922	0.0934
LGDHS and BSS	6	0.999	0.682
LKYDS and BSS	4	0.988	0.44
SDDES and LGDHS	5	0.999	0.549
